# The influence of future self-continuity on the willingness to engage in self-satisfied consumption: the mediating role of perceived economic mobility

**DOI:** 10.3389/fpsyg.2025.1728540

**Published:** 2026-01-16

**Authors:** Mingli Shi, Chunlei Fan, Ting Tao, Wenbin Gao

**Affiliations:** 1 Institute of Psychology, Chinese Academy of Sciences, Beijing, China; 2 Department of Psychology, University of Chinese Academy of Sciences, Beijing, China

**Keywords:** future self-continuity, intertemporal decision-making, mediation effect, perceived economic mobility, self-satisfied consumption

## Abstract

**Introduction:**

With the rise of emotional-value-driven consumption, self-satisfied consumption has become a significant market trend. However, research on its underlying psychological mechanisms remains limited. This study focuses on its future-oriented aspect, investigating how future self-continuity and perceived economic mobility influence the intention for self-satisfied consumption.

**Methods:**

A questionnaire-based study was conducted, collecting 388 valid responses nationwide. The concept of self-satisfied consumption was synthesized as healthy, rational, and autonomous consumption driven by self-development and personal pleasure. Data were analyzed using Hayes’ SPSS Process macro (Model 4) to test the proposed mediation model.

**Results:**

(1) Both future self-continuity and perceived economic mobility positively predicted the intention for self-satisfied consumption. (2) Marital status was a significant demographic factor, with married individuals showing higher intention than unmarried ones. (3) Perceived economic mobility played a significant partial mediating role in the relationship between future self-continuity and consumption intention.

**Discussion:**

The findings confirm that future-oriented psychological factors are key drivers of self-satisfied consumption, revealing a specific pathway through perceived economic mobility. This extends the theoretical understanding of the formation mechanisms of self-satisfied consumption and offers practical insights for consumer psychology and marketing strategies aimed at enhancing consumer well-being.

## 1 Introduction

According to the 2024 Double 11 Consumer Population White Paper, emotional value has become a new consumption driver in recent years. Consumers are willing to pay for products with comforting features and self-satisfying experiences, such as blind boxes, stress-relief squeezable toys, and Capybara plush toys. They seek not only the product itself but also the emotional connections that bring joy, relaxation, and companionship. This consumption trend reflects people’s higher pursuit of life quality and reveals the increasingly close link between emotion and consumption (Damopan and Alimama Marketing Research Center, 2024). iiMedia Research (2024) Survey Data on “Interest-driven Consumption” Behavior of Chinese New Youth indicates that satisfying the self accounts for 46.28% of youth consumption (iiMedia Research, 2024). Meanwhile, data from the 2025 Taobao 618 shopping festival show that the collectible toy industry, where self-satisfied elements are prominent, captured 70 % of the market share, with its transaction scale ranking first for the consecutive year (Jieshu Consulting, 2025).

These signs indicate that with the continuous development of the socio-economy and consumption culture, “self-satisfied consumption” is emerging as a new consumption trend, gradually occupying an increasing proportion of our consumer lives.

Currently, there is no completely unified academic definition for the concept of self-satisfied consumption. However, different scholars have offered their perspectives in various fields of research. For instance, Pan (2019) and Zhao (2025) have provided similar definitions of self-satisfied consumption from the perspective of consumption motivation. Pan Helin argues that “self-satisfied consumption” occurs when consumers spend to satisfy their own needs and enhance their happiness; Zhao Ruiling posits that it is a consumption form centered on fulfilling self-emotional needs, obtaining pleasure and emotional value through spending. Zhou (2019) and Qin (2025), in their definitions, have more elaborately addressed the composition of self-satisfied consumption. For example, Zhou Changcheng suggests that the current “self-satisfied consumption” in China can be roughly categorized into two types: “instant self-satisfied consumption” and “developmental self-satisfied consumption.” These two types differ in their ultimate timeframes and behavioral mechanisms. Qin Xinyuan also proposes that self-satisfied consumption mainly refers to economic activities aimed at satisfying individuals’ deep-seated spiritual needs and life enjoyment. The keyword of self-satisfied consumption is “self-satisfaction.” Unlike “satisfying others,” which involves following trends and seeking superficial satisfaction during consumption, the core of “self-satisfaction” lies in the attention to one’s inner emotions. Whether it is instant consumption to satisfy immediate emotional needs or developmental consumption focused on long-term improvement, both essentially fall within the scope of self-satisfied consumption.

Li (2021) and Huang (2020) have proposed different views by analyzing the basic characteristics of self-satisfied consumption behavior. Li Jiayuan suggests that “self-satisfied consumption” has four major characteristics: 1. The main consumer group is the relatively young, actively single demographic; 2. The standard for consumption value is more subjective rather than objective, reflecting greater personalization; 3. Consumption motivation shows a trend of diversification, primarily satisfying one’s own spiritual needs while also considering aspects like quality and utility; 4. It encompasses both the satisfaction of present desires and expectations for future life. Huang Shiyuan proposes that when determining whether a consumption behavior belongs to self-satisfied consumption, it must simultaneously satisfy three key characteristics: a sense of pleasure, autonomy, and meaningfulness.

By reviewing the definitions and explanations of self-satisfied consumption in the existing literature and synthesizing the key characteristics of self-satisfied consumption behavior, this study integrates the concept of self-satisfied consumption, defining it as: Self-satisfied consumption is a healthy, rational, and autonomous consumption based on the needs for self-development and self-satisfaction. It is less influenced by others and societal trends, and when selecting products or services that bring personal satisfaction, consumers are often price-insensitive.

Current research on self-satisfied consumption mainly focuses on its development direction and its impact on people’s lives, while studies on the mechanisms influencing self-satisfied consumption are still relatively few. Referring to the definition of self-satisfied consumption, a portion of it is based on the component of self-development consumption. Self-development is often future-oriented; therefore, we believe that self-satisfied consumption involves a certain degree of future-oriented consumption behavior aimed at achieving long-term goals. Based on this, we hypothesize that the relationship between an individual’s current self and future self might be an influencing factor for self-satisfied consumption behavior. Consequently, we have selected two variables that we hypothesize may impact the intention for self-satisfied consumption—future self-continuity and perceived economic mobility—to conduct this research.

Future self-continuity refers to an individual’s perception of the connectedness between their current and future self (Rutchick et al., 2018). It reflects a cognitive assessment of the continuity between one’s present and future identity across time (Urminsky, 2017). Research indicates that future self-continuity influences intertemporal decision-making, savings behavior, academic effort, ethical behavior, and health-related actions, thereby promoting long-term-oriented behaviors (Liu et al., 2018). Time discounting describes the phenomenon where the subjective value of future outcomes decreases as the delay until their receipt increases (Green et al., 1994). Individuals with high future self-continuity tend to exhibit lower time discount rates and are more inclined to choose rational, long-term beneficial options in intertemporal decisions. In contrast, those with lower future self-continuity demonstrate higher time discount rates, leading to a stronger preference for immediate but smaller rewards, often at the expense of greater long-term benefits. Existing literature has shown that future self-continuity promotes behaviors such as exercising, saving, and studying (Rutchick et al., 2018). It enhances individuals’ focus on their future self, narrows the psychological gap between the present and future self, and positively reinforces long-term behavioral outcomes (Rutchick et al., 2018). Individuals with high future self-continuity are more concerned about their future and perceive their future self as closer to their current self (Bryan and Hershfield, 2012).

Perceived economic mobility refers to an individual’s belief in their ability to improve their economic status through personal effort (Davidai and Gilovich, 2015; Yoon and Kim, 2016). As a subjective perception of upward economic mobility and improved financial conditions, it significantly influences individuals’ psychological adjustment and decision-making (Bak and Yi, 2020; Mani et al., 2013).

Studies suggest that individuals with high perceived economic mobility are less influenced by materialism, exhibit greater rationality in consumption, and demonstrate sound financial management behaviors (Szendrey and Fiala, 2018; Yoon and Wong, 2014). Although delayed rewards in intertemporal decisions may evoke uncertainty, individuals with high perceived economic mobility tend to counter the effect of time discounting through their positive outlook on the future and higher risk tolerance (Bak and Yi, 2020). Other research indicates that these individuals also report higher life satisfaction (Kwon et al., 2022). This positive mindset reduces their sensitivity to temporal distance, leading to a stronger focus on the future and a preference for larger delayed rewards (Huang et al., 2016). Conversely, individuals with low perceived economic mobility may exhibit impulsivity and short-sightedness due to negative emotional states, showing a stronger preference for smaller immediate gains (Chen and He, 2014). According to self-regulation theory, long-term goals are attainable through self-regulatory processes. When individuals believe that their current efforts are linked to future outcomes, they demonstrate greater persistence in challenging tasks (Baumeister and Vohs, 2007; Browman et al., 2019).

## 2 Research hypotheses

As a new form of consumption, the motivation behind self-satisfied consumption is not solely driven by individuals’ immediate desires but also, to some extent, stems from a positive anticipation of their future state and a desire to achieve personal satisfaction and growth through consumption (Zhou, 2019).

Future self-continuity, as an individual’s perception of the relationship between their current and future self, is hypothesized to positively influence the willingness to invest in one’s future. We propose that the higher an individual’s sense of future self-continuity, the stronger their intention to engage in behaviors that benefit their future self. Similarly, perceived economic mobility, which reflects one’s belief in the possibility of future economic improvement and their sense of control over their economic trajectory, is also expected to promote future-oriented investment. Thus, we hypothesize that both future self-continuity and perceived economic mobility can positively predict the intention to engage in self-satisfied consumption.

Furthermore, we speculate that individuals with higher future self-continuity are more willing to make present efforts to secure greater future benefits. These efforts, in turn, may enhance their sense of control over future economic outcomes, thereby strengthening perceived economic mobility. Consequently, we further hypothesize that future self-continuity may indirectly promote self-satisfied consumption through its positive influence on perceived economic mobility. That is, perceived economic mobility may partially mediate the relationship between future self-continuity and self-satisfied consumption intention.

## 3 Research subjects and methods

### 3.1 Research subjects

From October 29 to November 24, 2021, a total of 582 samples were collected from 132 cities across China through the Credamo platform with monetary compensation. Based on attention-check questions, response duration, validation item answers, age, and other criteria, 94 invalid responses were excluded, resulting in a final valid sample of 388 participants.

Given that the measurement of self-satisfied consumption is related to price sensitivity and considering that the mechanisms or influencing factors of price sensitivity may differ across income groups, this study focused on middle-income individuals to enhance sample homogeneity. Referring to the definition of middle-income groups in the “Social Blue Book: Analysis and Forecast of Chinese Society in 2020,” the target population was defined as those with an annual household income between CNY 100,000 and 500,000. Screening was applied through the platform settings accordingly.

To exclude invalid responses, a validation item was included in the questionnaire: “My future economic status may be related to my current situation. This question is used to check your response attention. Please select zero. (0 means it entirely depends on my background; 10 means it entirely depends on what I do now).” Samples that did not select “0” were deemed invalid. Additionally, since this study focuses on self-satisfied consumption, the target age range was set between 24 and 43 years. The primary reason for selecting this age group is that Chinese university graduates are typically between 22 and 24 years old. Therefore, we set the minimum age of our study participants at 24 and defined the age range for this research as the first two decades after entering the workforce. Responses from participants outside this age range were also considered invalid and excluded.

### 3.2 Self-satisfied consumption questionnaire

As shown in Table 1, the self-satisfied consumption questionnaire was adapted from Huang (2020) from Beijing Normal University. This questionnaire was developed with reference to the item structure of the Status Consumption Scale (Eastman et al., 1999) and the Experiential Buying Tendency Scale (Howell et al., 2012). The item content was adapted based on insights from market research reports and tailored to align with the key characteristics of self-pleasure consumption. The questionnaire consists of 9 items, including 7 positively scored questions and 2 reverse-scored questions. All items were measured on a 5-point Likert scale, and the average score of the total questionnaire was used to represent the level of self-satisfied consumption intention.

**Table 1:** Self-satisfied consumption questionnaire.

Questions	1	2	3	4	5
When making purchases, I try to please myself to the greatest extent within my means.					
It is unimportant to me whether a product/service enhances my quality of life. (R)					
I am willing to pay slightly more for a product/service if it provides positive physical or emotional benefits.					
I would purchase a product/service solely because it enhances my happiness.					
I am willing to pay slightly more for a product/service if it makes me feel comfortable and satisfied.					
In daily consumption, I judge the value of what I buy based on my own criteria, with little regard for others’ opinions.					
I am willing to pay slightly more for a product/service if it contributes to my self-improvement and development.					
When consuming, “others’ appreciation” matters more to me than “personal preference.” (R)					
I would purchase a product/service simply because it brings me joy.					

All items were rated on a 5-point Likert scale from 1 (Strongly disagree) to 5 (Strongly agree). (R) indicates a reverse-scored item.

### 3.3 Future self-continuity scale

For the measurement of future self-continuity, many previous studies have adopted the scale developed by Ersner-Hershfield et al. (2009). This scale uses the degree of overlap between two circles to represent the continuity between the “current self” and the “future self.” Among the seven images in the scale, a higher degree of overlap indicates a stronger connection between the present and future self, reflecting greater future self-continuity. However, this scale has certain limitations: it is relatively simplistic, comprising only a single-dimensional measurement. Moreover, as it was originally designed for English-speaking populations, its translated version may exhibit some biases when applied in a Chinese context.

Therefore, this study employed another Future Self-Continuity Scale developed by Li (2019). This scale consists of 8 items (see Table 2), with the third item being reverse-scored. Responses were measured on a 7-point Likert scale, and the average score of the total items was used to represent the level of future self-continuity.

**Table 2:** Future self-continuity scale.

Questions	1	2	3	4	5	6	7
I look forward to seeing who I will become in 10 years.							
I feel that who I am now is the foundation for who I will be in 10 years.							
In fact, who I am in 10 years has little to do with who I am now.(R)							
I really like the person I imagine myself to be in 10 years.							
I believe my current actions will influence my development in 10 years.							
I feel excited about the person I imagine myself to be in 10 years.							
I feel a connection between who I am today and who I will be in 10 years.							
When I think about myself in 10 years, I feel a sense of aspiration.							

All items were rated on a 7-point Likert scale from 1 (Strongly disagree) to 7 (Strongly agree). (R) indicates a reverse-scored item. The total score was calculated by averaging responses across all items, with higher scores indicating stronger future self-continuity.

### 3.4 Perceived economic mobility questionnaire

Perceived economic mobility was measured using a two-item scale (Yoon and Kim, 2016). As presented in Table 3, participants rated their beliefs on an 11-point continuum (0 to 10) between two contrasting statements. The average score of the two items was calculated to form a composite index of perceived economic mobility, with higher scores indicating a stronger belief that one’s future economic status is determined by internal factors (e.g., one’s own efforts) rather than external factors (e.g., background). In this study, the two items showed a significant positive correlation, r < 0.001, supporting the scale’s reliability.

**Table 3:** Items of the perceived economic mobility scale.

Item	Endpoint(0)	Endpoint(10)
1	My future economic status depends on external factors.	My future economic status depends on my own efforts.
2	My future economic status primarily depends on my background.	My future economic status primarily depends on what I am doing now.

Items were rated on an 11-point scale from 0 to 10. The composite score was calculated by averaging the responses to both items.

## 4 Results

### 4.1 Assessment of the measurement model

See Tables 4, 5.

**Table 4:** Reliability and convergent validity of the measurement models.

Scale	Cronbach‘s *α*	Composite reliability (Rho_a)	Composite reliability (Rho_c)	AVE
Self-satisfied consumption	0.718	0.754	0.825	0.547
Future self-continuity	0.844	0.852	0.885	0.530
Perceived economic mobility	0.843	0.843	0.927	0.864

**Table 5:** Discriminant validity assessment: Heterotrait-monotrait ratio (HTMT).

Scale	Self-satisfied consumption	Future self-continuity	Perceived economic mobility
Self-satisfied consumption	-	-	-
Future self-continuity	0.357	-	-
Perceived economic mobility	0.308	0.445	-

### 4.2 Control and examination of common method bias

As the research data were collected via questionnaires, the results might be influenced by common method bias. Harman’s single-factor test was employed to statistically assess common method bias (An et al., 2020). The first factor explained 24.289% of the total variance, which is below the recommended threshold of 40%. Therefore, no significant common method bias was detected in this study.

### 4.3 Demographic variables

Three demographic variables were included in the questionnaire: gender, age, and marital status. The specific distribution of the sample data is presented in Table 6.

**Table 6:** Description of demographic information.

Demographic variable	Category	Sample size
Gender	Male	182
	Female	206
Marital Status	Married	200
	Unmarried	188
Age	24–33	219
	34–43	169

### 4.4 Comparative analysis of variables by binary grouping

#### 4.4.1 Age grouping

To examine age-related differences, participants were divided into two groups using the median age of 33 years as a cutoff. Samples with an age ≤ 33 were classified as the younger group, while those older than 33 were classified as the older group. An independent samples t-test was conducted with self-satisfied consumption intention as the dependent variable.

As shown in Table 7, there was a significant difference in self-satisfied consumption intention between the younger group and the older group. The mean score of self-satisfied consumption intention in the older group was significantly higher than that in the younger group. This finding further supports the results of the correlation analysis in this study, indicating that within the investigated age range (24–43 years), age can positively predict self-satisfied consumption intention.

**Table 7:** Age group comparison.

Scale	Sample size	Self-satisfied consumption(M)	SD	t	*p*
Younger Group (24–33 years)	219	3.97	0.48	−2.354	0.019*
Older Group (34–43 years)	169	4.09	0.45		

*p < 0.05 (two-tailed). M, Mean.

#### 4.4.2 Gender grouping

Samples were divided into groups based on gender, with the male group comprising 206 samples and the female group comprising 182 samples. An independent samples t-test was performed on the two gender groups, and the results are presented in Table 8.

**Table 8:** Gender group comparison.

Group	Sample size	Self-satisfied consumption(M)	SD	t	*p*
Male	206	4.02	0.50	−0.226	0.821
Female	182	4.03	0.45		

M, Mean.

As shown in the results of Table 8, no significant difference in self-satisfied consumption intention was observed between the male and female groups, indicating that gender does not exert a significant influence on self-satisfied consumption intention.

#### 4.4.3 Marital status grouping

Participants were divided into groups based on marital status, with the unmarried group comprising 188 samples and the married group comprising 200 samples. An independent samples t-test was conducted to compare the two groups, and the results are presented in Table 9. As shown in the table, a significant difference in self-satisfied consumption intention was found between the unmarried and married groups.

**Table 9:** Marital status group comparison.

Group	Sample size	Age(M ± SD)	Self-satisfied consumption(M)	SD	T	*p*
Unmarried group	188	33.08 ± 2.62	3.94	0.48	−3.331	0.001^***^
Married group	200	34.37 ± 2.99	4.10	0.45		

***p ≤ 0.001(two-tailed). M, Mean.

The mean score of self-satisfied consumption intention in the married group was significantly higher than that in the unmarried group. This indicates that marital status has a significant impact on self-satisfied consumption intention, with unmarried individuals exhibiting a higher level of self-satisfied consumption intention compared to married individuals.

When compared with the age grouping results, both age and marital status appear to significantly influence self-satisfied consumption. However, since the effects of age and marital status on self-satisfied consumption may overlap, a comparison of the average age between the unmarried and married groups was conducted. The results revealed a significant difference in age between the two groups (*t* = −4.353, *p* < 0.001). This suggests that as age increases, the likelihood of marriage also rises significantly. Therefore, age may influence self-satisfied consumption indirectly through its effect on marital status.

To further clarify the respective influences of age and marital status on self-satisfied consumption intention, a hierarchical regression analysis was conducted to determine the layered effects of these two variables. It is generally believed that the relationship between age and marital status is such that age acts as a cause of driving changes in marital status. Thus, after controlling for the effect of marital status on self-satisfied consumption, analyzing the independent effect of age on self-satisfied consumption can help clarify their relational dynamics in influencing consumption intention. Accordingly, this study first conducted a regression analysis with marital status as the control variable (Layer 1), followed by a second regression analysis with age as the test variable (Layer 2).

The results, as shown in Table 10, indicate that after controlling for the effect of marital status on self-satisfied consumption, age was no longer significantly correlated with self-satisfied consumption intention (*t* = 1.348, *p* = 0.179, Beta = 0.069, ΔR^2^ = 0.005). This demonstrates that the influence of age on self-satisfied consumption is primarily mediated through marital status. When the effect of marital status is controlled, age does not exert a significant independent impact on self-satisfied consumption.

**Table 10:** Hierarchical regression analysis of age and marriage on self-satisfied consumption willingness.

Variable	Step 1				Step 2			
	Beta	SE	t	*p*	Beta	SE	t	*p*
Marital status	0.167	0.047	3.337	0.001^***^	0.152	0.048	2.970	0.003^**^
Age	-	-	-	-	0.069	0.008	1.348	0.179
F	11.137				6.489			
R2	0.028				0.033			
Adjusted R2	0.026				0.028			
△R2	-				0.005			

**p ≤ 0.01, ***p ≤ 0.001 (two-tailed).

#### 4.4.4 Future self-continuity and perceived economic mobility grouping

The data were median grouped in ascending order based on scores of future self-continuity and perceived economic mobility. Under the premise that samples with the same score were assigned to the same group, the participants were divided into low-score and high-score groups for each variable, ensuring that the groups were as equal in size as possible. The specific grouping results are as follows:

For future self-continuity:

- Low-score group (*N* = 194, M = 5.00, SD = 0.67)
- High-score group (*N* = 194, M = 6.37, SD = 0.32)

For perceived economic mobility:

- Low-score group (*N* = 209, M = 5.19, SD = 1.13)
- High-score group (*N* = 179, M = 8.65, SD = 0.88)

After grouping, independent samples t-tests were conducted to compare the self-satisfied consumption intention between the low-score and high-score groups for each independent variable. The results of these analyses are presented in Table 11.

**Table 11:** Future self-continuity, perception of economic mobility group comparison.

Variable	Group name	Self-satisfied consumption(M)	SD	t	*p*
Future self-continuity	Low-score group(*N* = 194)	3.91	0.43	−5.00	<0.001^***^
	High-score group(*N* = 194)	4.14	0.48		
Perceived economic mobility	Low-score group(*N* = 209)	3.95	0.45	−3.157	0.002^**^
	High-score group(*N* = 179)	4.10	0.49		

**p ≤ 0.01 (two-tailed), ***p ≤ 0.001(two-tailed).

The test results indicate that after grouping the independent variables—future self-continuity and perceived economic mobility—into low-score and high-score groups based on ascending values, significant differences in self-satisfied consumption intention were observed between the low-score and high-score groups for both variables. These findings further validate the correlation between these variables and self-satisfied consumption.

### 4.5 Correlation analysis

See Table 12.

**Table 12:** Correlation analysis of future self-continuity, perception of economic mobility, and self-satisfied consumption correlation analysis.

Variable		1	2	3
1 Future Self-Continuity	*r*	-		
	*p*			
2 Perceived Economic Mobility	*r*	0.384	-	
	*p*	<0.001^***^		
3 Self-Satisfied Consumption	*r*	0.326	0.237	
	*p*	<0.001^***^	<0.001^***^	-

***p ≤ 0.001(two-tailed).

#### 4.5.1 Effects of future self-continuity on self-satisfied consumption

Consistent with previous research on the influence of future self-continuity on intertemporal decision-making, individuals with stronger future self-continuity tend to reduce the time discounting effect, enabling them to allocate weights more rationally between present and future gains and losses. As a result, they are more inclined to invest in themselves to pursue better future development (Tan, 2020). Since self-satisfied consumption inherently includes aspects oriented toward future development, future self-continuity can positively predict self-satisfied consumption intention.

#### 4.5.2 Effects of perceived economic mobility on self-satisfied consumption

As another form of individual assessment of their future self, perceived economic mobility often reflects one’s sense of control over their future socioeconomic status. Higher perceived economic mobility indicates a stronger sense of control over future economic capabilities and more optimistic expectations regarding future financial capacity. This tends to alter existing savings or investment habits, leading to a greater inclination to reduce savings and increase investment (Szendrey and Fiala, 2018). From this perspective, the relationship between perceived economic mobility and self-satisfied consumption intention can be further explained.

#### 4.5.3 Hierarchical regression analysis of future self-continuity on self-satisfied consumption

To eliminate potential confounding effects of control variables in the relationship between future self-continuity and self-satisfied consumption intention, this study conducted a hierarchical regression analysis to examine the independent influence of future self-continuity on self-satisfied consumption intention.

In the hierarchical regression analysis, self-satisfied consumption was set as the dependent variable. The first layer of regression included gender, age, and marital status as control variables, while the second layer introduced future self-continuity as the independent variable.

The results of the two-layer regression analysis, as shown in Table 13, indicate that after controlling for the effects of gender, age, and marital status, future self-continuity continued to positively predict self-satisfied consumption intention (*t* = 6.488, *p* < 0.001, Beta = 0.314, ΔR^2^ = 0.096). This confirms the independent positive predictive effect of future self-continuity on self-satisfied consumption.

**Table 13:** Hierarchical regression analysis of future self-continuity on self-satisfied consumption.

Variables	Step 1				Step 2			
	B	SE	t	*p*	B	SE	t	*p*
Gender	−0.004	0.048	−0.076	0.940	−0.028	0.046	−0.578	0.564
Age	0.07	0.008	1.348	0.179	0.085	0.008	1.725	0.085
Marital status	0.152	0.049	2.945	0.003^**^	0.102	0.047	2.057	0.040^*^
Future self-continuity	-	-	-	-	0.314	0.026	6.488	<0.001^***^
F	4.317				14.107			
R^2^	0.033				0.128			
Adjusted R^2^	0.025				0.115			
△R^2^	-				0.096			

*p < 0.05 (two-tailed), **p ≤ 0.01 (two-tailed), ***p ≤ 0.001(two-tailed).

### 4.6 Analysis of the mediating role of perceived economic mobility

Building on the correlation analyses among future self-continuity, perceived economic mobility, and self-satisfied consumption, this study used Model 4 from Hayes’ SPSS Process 3.4 to conduct a regression analysis. Future self-continuity was set as the independent variable, self-satisfied consumption as the dependent variable, and perceived economic mobility as the mediating variable. Considering the previously identified influence of marital status on self-satisfied consumption, marital status was included as a covariate in this analysis to control its potential confounding effects.

The data processing procedure followed the mediation effect testing approach and utilized the Bootstrap method (with 5,000 resamples) to examine the mediation effect (Wen et al., 2004; Preacher and Hayes, 2008). Whether the 95% confidence interval included zero was used to determine the significance of the mediating path. If the interval included zero, the path was considered insignificant; otherwise, it was deemed significant. The results of this analysis are presented in the Table 14.

**Table 14:** Analysis of the mediating role of perceived economic mobility.

Path	Effect Size	Boot SE	Boot LLCI	Boot ULCI	*p*
Future self-continuity → self-satisfied consumption (c’)	0.1455	0.0283	0.0899	0.2011	< 0.001^***^
Future self-continuity → perceived economic mobility (a)	0.8551	0.1098			< 0.001^***^
Perceived economic mobility → self-satisfied consumption (b)					0.0258*
Indirect effect (a × b)	0.0234	0.0108	0.0024	0.0447	
Total effect (c)	0.1689	-	-	-	

Unstandardized coefficients (B) are reported. SE, Standard Error. Boot LLCI/ULCI, Bias-corrected bootstrap 95% lower/upper confidence interval (based on 5,000 samples). The total effect (c) was calculated as the sum of the direct effect (c’) and the indirect effect (ab) (i.e., 0.1455 + 0.0234 = 0.1689). ***p < 0.001, **p < 0.01, *p < 0.05.

**Figure 1:**
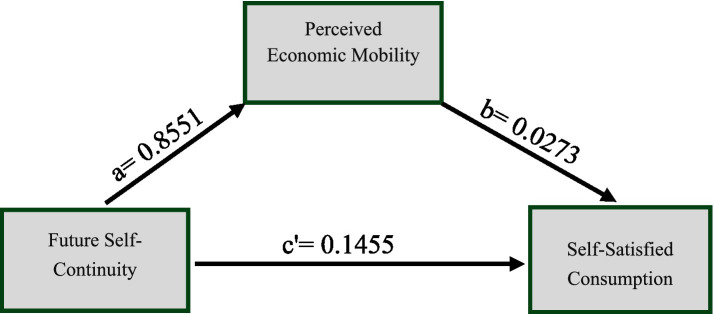
Mediation model of perceived economic mobility. Mediation model of perceived economic mobility. Unstandardized coefficients are shown. The model controlled for the effect of marital status. Indirect effect ab = 0.0234, 95% Boot CI [0.0024, 0.0447]. ***p < 0.001, *p < 0.05.

The analysis results (see Figure 1) indicate that, after controlling for the influence of marital status, future self-continuity still significantly and positively predicts perceived economic mobility (a = 0.8551, *p* < 0.001). Perceived economic mobility also significantly and positively predicts self-satisfied consumption intention (b = 0.0273, *p* = 0.026). The direct effect of future self-continuity on self-satisfied consumption intention remains significant (c’ = 0.1455, *p* < 0.001).

The analysis of the indirect effect revealed that the indirect effect of future self-continuity on self-satisfied consumption intention through perceived economic mobility was 0.0234, with a Bootstrap 95% confidence interval of [0.0024, 0.0447] (excluding zero). This indicates that the mediating effect of perceived economic mobility is statistically significant. In summary, these results demonstrate that perceived economic mobility plays a partial mediating role in the relationship between future self-continuity and self-satisfied consumption intention.

## 5 Conclusion

This study investigates the mechanisms influencing self-satisfied consumption, analyzing and validating the effects of demographic variables, future self-continuity, and perceived economic mobility on self-satisfied consumption.

This study yielded several key findings. First, regarding demographic factors, the influence of age on Self-satisfied Consumption intention was found to be primarily mediated by marital status, with married individuals exhibiting significantly higher levels than their unmarried counterparts. After controlling for marital status, no direct significant correlation between age and intention remained. Second, no significant correlation was observed between gender and Self-satisfied Consumption intention. More centrally to our hypotheses, both Future Self-continuity and Perceived Economic Mobility positively predicted Self-satisfied Consumption intention. Furthermore, Perceived Economic Mobility played a significant mediating role in the relationship between Future Self-continuity and Self-satisfied Consumption, elucidating a key psychological pathway.

This study examines the underlying mechanisms of Self-Satisfied Consumption, focusing on the impact of two future-oriented factors—Future Self-Continuity and Perceived Economic Mobility—on the intention for Self-Satisfied Consumption. Through a mediating model, it confirms that both Future Self-Continuity and Perceived Economic Mobility can directly enhance an individual’s intention for Self-Satisfied Consumption. Furthermore, Future Self-Continuity can also indirectly influence this intention by strengthening Perceived Economic Mobility. This finding reveals a facilitating mechanism through which Perceived Economic Mobility operates within the influence of Future Self-Continuity on personal Self-Satisfied Consumption intention. Based on the above findings, this research provides three insights for understanding the current consumer market: Firstly, specific attention should be paid to individuals who are married or in stable partnerships, as they tend to engage in moderate Self-Satisfied Consumption even during stressful periods to enhance family life quality and future planning. Designing products and services for this demographic is more likely to elicit market response. Secondly, businesses can focus on enhancing consumers’ two positive psychological cognitions—"Future Self-Continuity” and “Perceived Economic Mobility”—through brand communication and product design. For instance, shaping a positive and controllable future vision can be employed to boost consumption intention. Finally, following the pathway of “Future Self-Continuity → Perceived Economic Mobility → Self-Satisfied Consumption Intention,” it is possible to effectively promote Self-Satisfied Consumption behavior by simultaneously strengthening consumers’ sense of connection between their present and future selves and providing a clear, visible path for economic improvement.

While this study primarily investigates the impact of Future Self-Continuity and Perceived Economic Mobility on Self-Satisfied Consumption intention, several limitations remain, pointing the way for future research. First, regarding variable selection, this research mainly focused on Future Self-Continuity and Perceived Economic Mobility. However, against the backdrop of rapid economic and digital technological development, various emerging factors such as digital consumption, online reviews, social media, and big data may also influence consumption decisions. Future studies could incorporate a more diverse set of variables to more comprehensively reveal the formation mechanisms of Self-Satisfied Consumption intention. Second, in terms of sampling, this study recruited a nationwide sample covering over a hundred cities via online platforms. Given the economic, cultural, and educational disparities among different cities, these differences might impact group comparisons. Although key variables were controlled and balanced for in the study, future research could enhance the reliability and generalizability of results by designing stricter sampling procedures, expanding the sample size, and refining stratification. Third, methodologically, this study employed a cross-sectional design where all variables were measured at a single time point, making it difficult to establish causal relationships definitively. Although the theoretical model posited a pathway from Future Self-Continuity to Perceived Economic Mobility and then to Self-Satisfied Consumption intention, reverse causality or explanations involving third variables cannot be entirely ruled out. Future research could employ longitudinal tracking or experimental interventions (e.g., manipulating future orientation) to provide more robust evidence for causality. Fourth, concerning variable measurement and manipulation, this study treated Future Self-Continuity as a state variable for measurement and grouping. Subsequent research could systematically manipulate it experimentally to more precisely investigate its mechanism of action on Self-Satisfied Consumption intention. Furthermore, while this study found a significant impact of marital status on Self-Satisfied Consumption intention, the underlying mechanisms (e.g., cultural norms, social media usage) were not explored in depth, offering a promising avenue for future inquiry. Finally, regarding external validity, data collection occurred during the COVID-19 pandemic period. The unique social environment may have perturbed individuals’ future expectations and related psychological perceptions, thereby affecting the measurement results. Follow-up studies could compare data newly collected after the pandemic with the data from this phase to test the stability of the conclusions across different periods and to deeply explore the long-term impact of significant external events on consumption psychology.

Based on these considerations, future studies could conduct long-term tracking and replicate and expand this model using more diverse samples and additional research variables. Despite its limitations, this study offers new insights and a reference for further research on the formation mechanisms of self-satisfied consumption intention, contributing to the understanding of the factors that influence self-satisfied consumption.

## Data Availability

The raw data supporting the conclusions of this article will be made available by the authors, without undue reservation.
